# A review on the traditional uses, phytochemistry, and pharmacological activities of clove basil (*Ocimum gratissimum* L.)

**DOI:** 10.1016/j.heliyon.2021.e08404

**Published:** 2021-11-25

**Authors:** Ositadinma Chinyere Ugbogu, Okezie Emmanuel, Grace Oka Agi, Chibuike Ibe, Celestine Nwabu Ekweogu, Victor Chibueze Ude, Miracle Ebubechi Uche, Rachel Oluchukwu Nnanna, Eziuche Amadike Ugbogu

**Affiliations:** a Department of Microbiology, Abia State University, Uturu, PMB 2000, Uturu, Abia State, Nigeria; b Department of Biochemistry, Abia State University, PMB 2000, Uturu, Abia State, Nigeria; c Department of Human Nutrition and Dietetics, University of Ibadan, Nigeria; d Department of Medical Biochemistry, Imo State University, PMB 2000, Owerri, Imo State, Nigeria; e Department of Medical Biochemistry, College of Medicine Enugu State University of Science and Technology, PMB 01660, Enugu, Nigeria

**Keywords:** Essential oil, *Ocimum gratissimum*, Pharmacological activities, Phytochemicals, Traditional uses

## Abstract

In traditional medicine, *Ocimum gratissimum* (clove basil) is used in the treatment of various diseases such as diabetes, cancer, inflammation, anaemia, diarrhoea, pains, and fungal and bacterial infections. The present study reviewed the phytochemicals, essential oils, and pharmacological activities of *O. gratissimum*. The bioactive compounds extracted from *O. gratissimum* include phytochemicals (oleanolic acid, caffeic acid, ellagic acid, epicatechin, sinapic acid, rosmarinic acid, chlorogenic acid, luteolin, apigenin, nepetoidin, xanthomicrol, nevadensin, salvigenin, gallic acid, catechin, quercetin, rutin, and kaempfero) and essential oils (camphene, β-caryophyllene, α- and β-pinene, α-humulene, sabinene, β-myrcene, limonene, 1,8-cineole, trans-β-ocimene, linalool, α- and δ-terpineol, eugenol, α-copaene, β-elemene, p-cymene, thymol, and carvacrol). Various *in vivo* and *in vitro* studies have shown that *O. gratissimum* and its bioactive constituents possess pharmacological properties such as antioxidant, anti-inflammatory, anticancer, hepatoprotective, antidiabetic, antihypertensive, antidiarrhoeal, and antimicrobial properties. This review demonstrated that *O. gratissimum* has a strong preventive and therapeutic effect against several diseases. The effectiveness of *O. gratissimum* to ameliorate various diseases may be attributed to its antimicrobial and antioxidant properties as well as its capacity to improve the antioxidant systems. However, despite the widespread pharmacological activities of *O. gratissimum*, further experiments in human clinical trial studies are needed to establish effective and safe doses for the treatment of various diseases.

## 1 Introduction

The use of medicinal plants in traditional and complementary medicine for the treatment, management, or prevention of various diseases is as old as the origin of mankind (Yuan et al., 2016; Ekweogu et al., 2019). It has been estimated that approximately 80% of the world's population depends mainly on ethnomedicine or herbal medicine for the treatment of numerous diseases worldwide (Joshi, 2013; Pant, 2014). Interestingly, the increasing preference for the use of herbal medicines over conventional medicines may be attributed to the efficacies of the active ingredients present in herbal medicine to serve as natural healing agents as well as their availability, accessibility, affordability, and acclaimed less or non-toxic effects (Ikpeazu et al., 2018; Ijioma et al., 2021). Furthermore, over the last decade, medicinal plants and their bioactive compounds have attracted the attention of several researchers because of their usefulness in the management and prevention of life-threatening and chronic diseases (Sofowora et al., 2013; WHO, 2019) such as cancer, diabetes, stroke, and arthritis (Bernell and Howard, 2016), as an alternative therapy for the treatment of psychiatric disorders (Venuprasad et al., 2014), and in meeting the health requirements of the elderly (WHO, 2019). Currently, these medicinal plants have not only been employed in the treatment of numerous ailments, but also serve as a source of novel drugs for use in traditional or orthodox medicine. Drugs such as quinine, digoxin, aspirin, and morphine were produced from medicinal plants such as *Cinchona officinalis*, *Digitalis purpurea*, *Saix alba*, and *Papaver somniferum*, respectively (Mbanaso et al., 2020).

*Ocimum gratissimum L*., popularly known as scent leaf, is one of the discovered medicinal plants with the potential to serve as an alternative therapy for the treatment of various ailments or as a source of a new drug. It is a widespread and commercially viable perennial herbaceous plant with a very strong aromatic smell. It belongs to the family of Lamiaceae and is found in Africa, Asia, and South America (Tanko et al., 2008; Akara et al., 2021). It is used as a natural flavouring agent, condiment, or vegetable in the preparation of fish, meat, soup, and stew. It is also used in traditional medicine for the treatment of several ailments such as cough, pneumonia, fever, inflammation, anaemia, diarrhoea, pains, and fungal and bacterial infections (Akara et al., 2021).

Scientific reports have shown that *O. gratissimum* has a wide range of bioactive compounds such as flavonoids and polyphenols (Venuprasad et al., 2014; Irondi et al., 2016) and essential oils with several beneficial effects (Benitez et al., 2009; Melo et al., 2019), as shown in Tables 1 and 2.

**Table 1:** Chemical structures and biological activities of compounds isolated from *O. gratissimum*.

Name of Compound	Structure of compound	Method of identification	Biological activities/beneficial effects	References
Sinapic acid		LC-ESI–MS/MS of 70% ethanolic fraction of *O. gratissimum*Venuprasad et al. (2014)	Exhibits antioxidant, anti-inflammatory, anticancer, antimutagenic, antiglycemic, neuroprotective, and antibacterial activities.	Chen (2016)
Rosmarinic acid		LC-ESI–MS/MS of 70% ethanolic fraction of *O. gratissimum*Venuprasad et al. (2014)	Anti-microbial, immunomodulatory, anti-diabetic, anti-allergic, anti-inflammatory, hepato- and renal-protectant agent	Alagawany et al. (2017)
Luteolin		LC-ESI–MS/MS of 70% ethanolic fraction of *O. gratissimum*Venuprasad et al. (2014), High Performance Liquid Chromatography Coupled with Diode Array Detection (HPLC-DAD) using *O. gratissimum leaf extract* (Irondi et al., 2016)	antihypertension, anti-inflammatory, and anti-cancer.	Lin et al. (2008)
Apigenin		LC-ESI–MS/MS of 70% ethanolic fraction of *O. gratissimum*Venuprasad et al. (2014)	Anti-inflammatory, antioxidant, antibacterial and antiviral activities and blood pressure reduction	Yan et al. (2017)
Nepetoidin		LC-ESI–MS/MS of 70% ethanolic fraction of *O. gratissimum*Venuprasad et al. (2014)	Anti-oxidant, anti-viral, anti-fungal and anti-bacterial effects, xanthine oxidase, nitric oxide inhibitor	Grayer et al. (2003), Tsai and Lee (2014),
Xanthomicrol		LC-ESI–MS/MS of 70% ethanolic fraction of *O. gratissimum*Venuprasad et al. (2014)	Antiangiogenic and anticancer agent	Abbaszadeh et al. (2014), Panahi et al. (2018), Ghazizadeh et al. (2020)
Nevadensin		LC-ESI–MS/MS of 70% ethanolic fraction of *O. gratissimum*Venuprasad et al. (2014)	Antioxidant activities, Selective inhibitor of human carboxylesterase 1 (hCE1).	Tsai and Yin (2008), Wang et al. (2018)
Salvigenin		LC-ESI–MS/MS of 70% ethanolic fraction of *O. gratissimum*Venuprasad et al. (2014)	Antitumor Potent neuroprotective	Noori et al. (2013) Rafatian et al. (2012)
Oleanolic acid		LC-ESI–MS/MS of 70% ethanolic fraction of *O. gratissimum*Venuprasad et al. (2014)	Antioxidant, anti-inflammatory, antiviral, and anti-diabetic effects	Sen (2020)
Gallic acid		High Performance Liquid Chromatography Coupled with Diode Array Detection (HPLC-DAD) using *O. gratissimum leaf extract* (Irondi et al., 2016)	Antioxidant, anti-inflammatory, and antineoplastic, hepatoprotective and antihyperglycaemic properties	Kahkeshani et al. (2019), Huang et al. (2016), Hassani et al. (2020)
Catechin		High Performance Liquid Chromatography Coupled with Diode Array Detection (HPLC-DAD) using *O. gratissimum leaf extract* (Irondi et al., 2016)	Anti-inflammatory and anticancer, Antibacterial, anti-hypertensive, and antioxidative activities	Musial et al. (2020), Fan et al. (2017)
Chlorogenic acid		High Performance Liquid Chromatography Coupled with Diode Array Detection (HPLC-DAD) using *O. gratissimum leaf extract* (Irondi et al., 2016)	Antioxidant activity, antibacterial, hepatoprotective, cardioprotective, anti-inflammatory, antipyretic, neuroprotective, anti-obesity, antiviral, anti-microbial, anti-hypertension	Naveed et al. (2018)
Caffeic acid		High Performance Liquid Chromatography Coupled with Diode Array Detection (HPLC-DAD) using *O. gratissimum leaf extract* (Irondi et al., 2016)	Antioxidant, anti-inflammatory and anticarcinogenic activity.	Ye et al. (2010), Espíndola et al. (2019)
Ellagic acid		High Performance Liquid Chromatography Coupled with Diode Array Detection (HPLC-DAD) using *O. gratissimum leaf extract* (Irondi et al., 2016)	Anti-atherogenic, anti-inflammatory, and neuroprotective effects.	Ríos et al. (2018)
Epicatechin		High Performance Liquid Chromatography Coupled with Diode Array Detection (HPLC-DAD) using *O. gratissimum leaf extract* (Irondi et al., 2016)	Antiangiogenic, anti-diabetic, antioxidant and anticancer effects	Abdulkhaleq et al. (2017)
Quercetin		High Performance Liquid Chromatography Coupled with Diode Array Detection (HPLC-DAD) using *O. gratissimum leaf extract* (Irondi et al., 2016)	Antidiabetic, anti-inflammatory, antioxidant, antimicrobial, anti-Alzheimer's, antiarthritic, cardiovascular, and wound-healing effects	Salehi et al. (2020)
Rutin		High Performance Liquid Chromatography Coupled with Diode Array Detection (HPLC-DAD) using *O. gratissimum leaf extract* (Irondi et al., 2016)	Antioxidant, cytoprotective, vasoprotective, anticarcinogenic, neuroprotective and cardioprotective activities	Javed et al. (2012), Richetti et al. (2011), Ganeshpurkar and Saluja (2017)
Kaempferol		High Performance Liquid Chromatography Coupled with Diode Array Detection (HPLC-DAD) using *O. gratissimum* leaf extract (Irondi et al., 2016)	Antioxidant, anti-inflammatory, antimicrobial, anticancer, cardioprotective, neuroprotective, antidiabetic, anti-osteoporotic, estrogenic/antiestrogenic, anxiolytic, analgesic and antiallergic activities.	Calderón-Montaño et al. (2011)

**Table 2:** Chemical structures and biological activities of compounds isolated from *O. gratissimum* essential oil.

Name of Compound	Structure of compound	Method of identification	Biological activities/beneficial effects	References
Camphene		GC–MS of microwave-assisted hydrodistillation extracted essential oils from *O. gratissimum* (Benitez et al., 2009), GC/FID and GC/MS of extraction from fresh aerial parts of *O. gratissimum* (Kpadonou Kpoviessi et al., 2014)	Antioxidant activity and superoxide radical inhibition, hypolipidemic action	Quintans-Junior et al. (2013) Vallianou et al. (2011)
α- & β- Pinene		GC–MS of microwave-assisted hydrodistillation extracted essential oils from *O. gratissimum* (Benitez et al., 2009), GC/FID and GC/MS of extraction from fresh aerial parts of *O. gratissimum* (Kpadonou Kpoviessi et al., 2014), GC–MS and GC/FID of *O. gratissimum* leaf (Melo et al., 2019)	Antiviral, inhibitory, anticoagulant, antitumor, antimicrobial, antimalarial, antioxidant, anti-inflammatory, anti-leishmania, and analgesic effects	da Silva et al. (2012), Zhou et al. (2004), Salehi et al. (2019)
Sabinene		GC–MS of microwave-assisted hydrodistillation extracted essential oils from *O. gratissimum* (Benitez et al., 2009), GC/FID and GC/MS of extraction from fresh aerial parts of *O. gratissimum* (Kpadonou Kpoviessi et al., 2014), GC–MS and GC/FID of *O. gratissimum* leaf (Melo et al., 2019)	Anti-inflammatory activity, management of dermatophytosis	Valente et al. (2013)
β-Myrcene		GC–MS of microwave-assisted hydrodistillation extracted essential oils from *O. gratissimum* (Benitez et al., 2009), GC/FID and GC/MS of extraction from fresh aerial parts of *O. gratissimum* (Kpadonou Kpoviessi et al., 2014), GC–MS and GC/FID of *O. gratissimum* leaf (Melo et al., 2019)	Anti-inflammatory and anti-catabolic effects	Rufino et al. (2015)
Limonene		GC–MS of microwave-assisted hydrodistillation extracted essential oils from *O. gratissimum* (Benitez et al., 2009), GC/FID and GC/MS of extraction from fresh aerial parts of *O. gratissimum* (Kpadonou Kpoviessi et al., 2014)	Anti-inflammatory, antioxidant, antinociceptive, anticancer, antidiabetic, antihyperalgesic, antiviral, and gastroprotective effects, relief of heartburn and gastroesophageal reflux	Vieira et al. (2018) Sun (2007).
1,8-Cineole		GC–MS of microwave-assisted hydrodistillation extracted essential oils from *O. gratissimum* (Benitez et al., 2009), GC/FID and GC/MS of extraction from fresh aerial parts of *O. gratissimum* (Kpadonou Kpoviessi et al., 2014), GC–MS and GC/FID of *O. gratissimum* leaf (Melo et al., 2019)	Mucolytic and spasmolytic action on the respiratory tract, anti-inflammatory and anti-oxidant, antinociceptive activity	Juergens (2014), Liapi et al. (2007), Santos and Rao (2000)
trans-β-Ocimene		GC–MS of microwave-assisted hydrodistillation extracted essential oils from *O. gratissimum* (Benitez et al., 2009), GC/FID and GC/MS of extraction from fresh aerial parts of *O. gratissimum* (Kpadonou Kpoviessi et al., 2014), GC–MS and GC/FID of *O. gratissimum* leaf (Melo et al., 2019)	Anticonvulsant activity, antifungal activity, antitumor activity	Bomfim et al. (2016), Sayyah et al. (2004)
Linalool		GC–MS of microwave-assisted hydrodistillation extracted essential oils from *O. gratissimum* (Benitez et al., 2009), GC/FID and GC/MS of extraction from fresh aerial parts of *O. gratissimum* (Kpadonou Kpoviessi et al., 2014), GC–MS and GC/FID of *O. gratissimum* leaf (Melo et al., 2019)	Antimicrobial and insect-repellent properties, anti-inflammatory activity, antihyperlipidemic, antidepressant, neuroprotective and anticancer properties	Beier et al. (2014), Peana et al. (2002), Pereira et al. (2018)
α- & δ-Terpineol		GC–MS of microwave-assisted hydrodistillation extracted essential oils from *O. gratissimum* (Benitez et al., 2009), GC/FID and GC/MS of extraction from fresh aerial parts of *O. gratissimum* (Kpadonou Kpoviessi et al., 2014), GC–MS and GC/FID of *O. gratissimum* leaf (Melo et al., 2019)	Inhibits the growth of tumour cells Antibacterial activity	Hassan et al. (2010) Li et al. (2015)
Eugenol		GC–MS of microwave-assisted hydrodistillation extracted essential oils from *O. gratissimum* (Benitez et al., 2009), GC/FID and GC/MS of extraction from fresh aerial parts of *O. gratissimum* (Kpadonou Kpoviessi et al., 2014), GC–MS and GC/FID of *O. gratissimum* leaf (Melo et al., 2019)	Antimicrobial, anti-inflammatory, analgesic and antioxidant.	Mohammadi Nejad et al. (2017), Barboza et al. (2018); Fujisawa and Murakami (2016).
α-Copaene		GC–MS of microwave-assisted hydrodistillation extracted essential oils from *O. gratissimum* (Benitez et al., 2009), GC/FID and GC/MS of extraction from fresh aerial parts of *O. gratissimum* (Kpadonou Kpoviessi et al., 2014)	Antioxidant and antigenotoxic features, Potential anticancer agent	Turkez et al. (2014)
β-Elemene		GC–MS of microwave-assisted hydrodistillation extracted essential oils from *O. gratissimum* (Benitez et al., 2009), GC/FID and GC/MS of extraction from fresh aerial parts of *O. gratissimum* (Kpadonou Kpoviessi et al., 2014)	Anti-inflammatory and antitumor effects	Xie et al. (2020) Li et al. (2010)
p-cymene		GC/FID and GC/MS of extraction from fresh aerial parts of *O. gratissimum* (Kpadonou Kpoviessi et al., 2014)	Analgesic and anti-inflammatory properties	Santana et al. (2011)
Thymol		GC/FID and GC/MS of extraction from fresh aerial parts of *O. gratissimum* (Kpadonou Kpoviessi et al., 2014)	Antiseptic, antibacterial, antifungal, anthelmintic, antiviral, antioxidant, expectorant, antispasmodic, carminative, diaphoretic, sedative, anti-rheumatic, and even anti-cancer, anti-hyperlipidemic and anti-hyperglycemic action	Tohidi et al. (2020), Salehi et al. (2018), Li et al. (2017), Codruta et al. (2020), Tariq et al. (2019), Schnitzler (2019)
Carvacrol		GC/FID and GC/MS of extraction from fresh aerial parts of *O. gratissimum* (Kpadonou Kpoviessi et al., 2014)	Antimicrobial, antioxidant, and anticancer, analgesic, antispasmodic, antiinflammatory, angiogenic, antiparasitic, antiplatelet, AChe inhibitory, insecticidal, antihepatotoxic and hepatoprotective activities	Sharifi-Rad et al. (2018), Baser (2008)
β-caryophyllene		GC/FID and GC/MS of extraction from fresh aerial parts of *O. gratissimum* (Kpadonou Kpoviessi et al., 2014)	Antioxidant, anti-inflammatory, anticancer, cardioprotective, hepatoprotective, gastroprotective, nephroprotective, antimicrobial, and immune-modulatory activity.	Machado et al. (2018), Fidyt et al. (2016)
α-humulene		GC/FID and GC/MS of extraction from fresh aerial parts of *O. gratissimum* (Kpadonou Kpoviessi et al., 2014), GC–MS and GC/FID of *O. gratissimum* leaf (Melo et al., 2019)	Anti-inflammatory properties	Rogerio et al. (2009)

Furthermore, several studies have shown this plant possesses numerous pharmacological properties such as anti-hyperglycaemic (Aguiyi et al., 2000; Casanova et al., 2014), hypoglycaemic (Shittu et al., 2019), anti-inflammatory (Ajayi et al., 2019), anti-diarrhoeal (Offiah and Chikwendu, 1999), anti-anaemic, hepatoprotective (Akara et al., 2021), anti-hypertensive (Shaw et al., 2017), antibacterial (Melo et al., 2019), antifungal (Mohr et al., 2017), and anti-oxidative properties (Joshi, 2013; Mahapatra and Roy, 2014) as well as exhibits many other pharmacological activities.

This study aims to provide comprehensive and up-to-date information on the medicinal uses, bioactive phytochemicals or essential oils, and the pharmacological activities of *O. gratissimum*. This paper also provides useful information on the beneficial effects of *O. gratissimum* and identifies gaps in current knowledge that can encourage further investigation into the effectiveness and commercialization of *O. gratissimum* in the treatment of various human diseases.

## 2 Methods used to obtain the materials for the review

In this study, only peer-reviewed journals and papers published in English were used. All the relevant materials were collected from only four online databases, namely PubMed (https://pubmed.ncbi.nlm.nih.gov/), Springer (https://www.springer.com/gp), Wiley (https://www.wiley.com/en-us), and Sciencedirect (https://www.sciencedirect.com/). The key search terms or words were *Ocimum gratissimum* or scent leaf alone or in combination with botanical description, taxonomy, ethnopharmacological uses, phytochemistry, essential oil, pharmacological activities, antioxidant activity, anxiolytic activity, antinociceptive activity, neuroprotective activity, anti-bacterial activity, anti-fungal activity, anti-viral activity, anti-protozoal activity, anti-anaemic activity, wound healing properties, and analgesic activity.

## 3 Botanical description, distribution, and taxonomy

### 3.1 Botanical description

*Ocimum gratissimum* is an aromatic herbaceous plant also known as basil, basil-clove, or alfavaca. It belongs to the Lamiaceae family (genus *Ocimum* and species *gratissimum*) (Nweze and Eze, 2009). It is about 1–3 cm tall, has an erect stem, and is branched, round-quadrangular, and woody at the base, with opposite, slender, and marginalized leaves.

### 3.2 Geographic location

*Ocimum gratissimum* is a perennial and odoriferous shrub found in tropical regions such as Brazil, India, Vietnam, Rwanda, Nigeria (Lahlou et al., 2004; Nweze and Eze, 2009), Cameroon, Togo, In Côte d'Ivoire, Kenya, Benin (Kpoviessi et al., 2014), and South Africa (Venuprasad et al., 2014).

### 3.3 Taxonomy

Plants of the Lamiaceae family are mostly classified as spices, herbs, and other aromatic variations. The Lamiaceae family comprises 236 genera and 7200 species of vines, shrubs, and trees found all over the globe. The genus *Ocimum* comprises about 60 species, most of which are found in Africa (Tanko et al., 2008). *Ocimum gratissimum* can be found in many forms and oftentimes classified into different species and subspecies.

### 3.4 Traditional uses

*Ocimum gratissimum* has been described as a plant easily available to communities and commonly utilized for the treatment of a plethora of diseases in numerous ethnopharmacological surveys (Ajayi et al., 2017a,b,c). This perennial and odoriferous plant is now found in all continents and possesses generally acknowledged medicinal properties. Its medicinal potential in Africa is incredibly vast and varies by country (Kpoviessi et al., 2014). Its infusions are regarded as tonic and pectoral in Cameroon, and the juice of its sheets is used to treat giddiness, headaches, cold, and cough. In Côte d'Ivoire, several formulations of this plant are used to treat ear infections, dermatoses, and ophthalmias (Kpoviessi et al., 2014). In Nigeria, it is recommended for diarrhoea therapy (Kpoviessi et al., 2014), while Sofowora (1970) recommended it for respiratory ailments and for use as an anthelminthic. It was also used to treat headaches, fevers, and ophthalmic and skin problems, as well as pneumonitis. In Togo, the plant's infusion is used to relieve cough (antitussive). The fresh juice from its leaves has antidiarrheic and antidysentric properties; and its aqueous maceration is used to treat haematuria and purulent urethritis (Kpoviessi et al., 2014). In Benin Republic, the aqueous maceration of its pulp or aerial portions is used to treat dystopias, pelvic aches, colic, candidoses, digestive dysmenorrhoea, emesis, haemorrhoid (pile), and diarrhoea. Its stem decoction is used to treat hepatitis, cough, asthma, and wound infections (Chah et al., 2006; Kpoviessi et al., 2014). The juice from its leaves is used to treat angina, cephalgias, headaches, fever, and malnutrition. Its inflorescences are utilized as aromatizers in a variety of meals.

Previous studies have shown that this type of basil has anaesthetic, anti-stress, anti-inflammatory, anthelminthic, antidiarrhoeal (Offiah and Chikwendu, 1999), antipyretic, anti-mutagenic, anti-ulcerative, gastroprotective, hepatoprotective, sedative, and fungicidal properties, validating its widespread medical use (Priyanka et al., 2018; Martins et al., 2021). *O*. *gratissimum* is antiseptic and has found widespread applications in the preparation of toothpaste and mouthwash and in topical therapies (Pessoa et al., 2002). It is an excellent wash for sore throat and tonsillitis. It is also used as an expectorant and as a cough suppressant. The plant extract is used to treat gastrointestinal helminths in both animals and humans (Chitwood, 2002). Reports on *O*. *gratissimum* revealed that the plant extract may be used as a medicinal resource for people living with the human immunodeficiency virus (HIV) and acquired immunodeficiency syndrome (AIDS) (Priyanka et al., 2018). It is used as a febrifuge and as a component in several malarial treatments in West Africa (Chah et al., 2006). Crushed leaves of the plant are used to cure conjunctivitis, while the oil extracted from the leaves is considered highly antimicrobial and has been used in wound dressing, the preparation of mouthwash, and the prevent postnatal sepsis (Chah et al., 2006). Furthermore, fresh aerial portions are consumed directly as vegetables in traditional soups, while dried and powdered aerial parts are utilized in a variety of traditional dishes (Kpoviessi et al., 2014).

## 4 Phytochemistry

### 4.1 Polyphenols and flavonoids found in *Ocimum gratissimum*

The phenolic compounds found in *Ocimum gratissimum* include rosmarinic acid, sinapic acid, salvigenin, gallic acid, catechins, methyl eugenol, caffeic acid, L-caftaric, ellagic acid, trans-ferulic acid, L-chicoric acid, and flavonoids such as xanthomicrol, cirsimaritin, rutin, apigenin, kaempferol, vicenin-2, luteolin 5-O-glucoside, luteolin 7-O-glucoside, 7,4,′-dimethyl ether, vitexin, isovitexin, nepetoidin A, quercetin 3-O-glucoside, nevadensin, cirsimaritin, hymenoxin, myricetin, basilimoside, morin, isothymusin (Grayer et al., 2000; Costa et al., 2012; Ouyang et al., 2013; Casanova et al., 2014; Venuprasad et al., 2014; Ajayi et al., 2019), epicatechin, quercitrin, quercetin (Irondi et al., 2016), and triterpenes (oleanolic, pomolic acid, ursolic acids, and tormentic acid) (Dzoyem et al., 2021). The bioactive phenolic compounds and flavonoids identified from *O. gratissimum* as well as their structures and various pharmacological activities are shown in Table 1.

### 4.2 Chemical constituents of essential oil present in *O. gratissimum*

Compounds present in the essential oil of *O. gratissimum* include hydrocarbonated monoterpenes such as camphene, α-thujene, α-pinene, sabinene, β-pinene, β-myrcene, α and β-phellandrene, δ-3-carene, limonene, α-terpinene, p-cymene, trans-β-ocimene, γ-terpinene, terpinolene, p-cymenene, and p-menthane-1,3,8-triene; oxygenated monoterpenes such as 1.8-cineole, cis-sabinene hydrate, linalool, trans-sabinene hydrate, trans-thujone, citronellal, umbellulone, borneol, terpinen-4-ol, p-cymen-8-ol, α-terpineol, thymol methyl ether, estragol, p-cymen-7-ol, thymol, and carvacrol; hydrocarbonated sesquiterpenes such as α-copaene, β-elemene, γ-elemene, β-caryophyllene, α-trans-bergamotene, α-humulene, β-bourbunene, α-guaiene, δ-cadinene, germacrene D, γ-selinene, β-selinene, α-selinene, (Z,E)-α-farnesene, and 7-epi-α-selinene; and oxygenated sesquiterpenes such as caryophyllene oxide, 1,2-epoxydehumulene, and 3,7-(11)-eudesmadiene, spathulenol (Vieira et al., 2001; Pessoa et al., 2002; Lahlou et al., 2004; Tchoumbougnang et al., 2005; Lemos et al., 2005; Benitez et al., 2009; Kpoviessi et al. 2012, 2014; Nguemtchouin et al., 2013; Aguiar et al., 2015; Mohr et al., 2017; Chimnoi et al., 2018; Melo et al., 2019; Onyebuchi and Kavaz, 2020; Essoung et al., 2020). The bioactive compounds identified from the essential oil of *O. gratissimum* as well as their structures and various biological activities are shown in Table 2.

## 5 Pharmacological activities

### 5.1 Antioxidant activity

The antioxidant and anti-inflammatory properties of *Ocimum gratissimum* have been ascribed to its therapeutic benefits (Olamilosoye et al., 2019; Oyem et al., 2021). Its leaf extracts have been shown to contain antioxidant vitamins such as alpha-tocopherol and ascorbic acid (Olamilosoye et al., 2019). Previous research has shown that flavonoids and phenols protect against oxidative stress-induced cellular damage. Flavonoids and phenols exert anti-inflammatory and anti-oxidative effects through a variety of mechanisms, such as scavenging or quenching free radicals, chelating metal ions, or blocking enzyme systems that generate free radicals Olamilosoye et al., (2019). The presence of saponins, terpenoids, glycosides, and alkaloids in the aqueous extract of *O*. *gratissimum* may further contribute to its anti-inflammatory and anti-oxidative activities (Olamilosoye et al., 2019; Oyem et al., 2021). Joshi (2013) investigated the antioxidant activities of *O*. *gratissimum* and eugenol essential oils utilizing 2,2-diphenyl-1-picrylhydrazyl (DPPH) and 2,2′-azino-bis (3-ethylbenzothiazoline-6-sulfonic acid (ABTS) test models. The DPPH and ABTS models had substantial IC_50_ values of 23.66 and 23.91, respectively, indicating that the essential oils of *O*. *gratissimum* may have antioxidant properties. The authors also revealed that the oils of *O*. *gratissimum* had higher antioxidant properties than pure eugenol (Joshi, 2013). Venuprasad et al. (2014) investigated the antioxidant activity of the leaf extract using a variety of *in vitro* free radical scavenging tests. The DPPH, iron chelating, ABTS, NO, and hydroxyl radical scavenging capabilities were tested, and the IC_50_ values were 470, 17.4, 133, 83, and 260 g/mL, respectively. The analytical results revealed that *O*. *gratissimum* may have free radical scavenging properties. Shittu et al. (2016) investigated the effects of the aqueous leaf extract of *O*. *gratissimum* on haematological and oxidative stress markers in alloxan-induced diabetic rats. The results showed that diabetic rats fed with the aqueous extract of *O*. *gratissimum* had a considerable decline in fasting blood glucose levels compared with untreated diabetic rats. There was a reversal of weight loss found in the rats tested. The authors also reported a decreased level in malondialdehyde (MDA) concentration and this marker increases during lipid peroxidation (Shittu et al., 2016) (Table 3).

**Table 3:** Summary of the effects of *Ocimum gratissimum* on different experimental models.

Doses	Experimental models	Observation	Effects	References
0.015–8.00 mg/ml essential oil extract of *O. gratissimum* leaf	Bacteria	Demonstrated rapid killing of *E. coli* and *S. Typhimurium*	Antimicrobial activity	Chimnoi et al. (2018)
16 μL essential oil extract of *O. gratissimum* leaf	Bacteria	Reduced the growth level of *S. aureus* and *E. coli*	Antimicrobial activity	Melo et al. (2019)
0.5ml of 80% ethanolic *O. gratissimum* leaf extract	Bacteria	The extract was active against *E. coli*, *P. aeruginosa*, *S. aureus*, and *B. cereus*	Antimicrobial activity	Talabi and Makanjuola et al. (2017)
1 ml of 0.009–5.0 mg/ml of *O. gratissimum* essential oil in prepared with 10% DMSO	Bacteria	The essential oil was highly active against *E. coli, S. marcescens*, and *K. pneumoniae*	Antimicrobial activity	Joshi (2013)
50 mg/ml,25 mg/ml,12.5 mg/ml and 6.25 mg/ml ethanolic extract of *O. gratisimum* leaf	Bacteria	*In vitro* activities against *E. coli, P. mirabilis, S. aureus* and *P. aeruginosa*	Antimicrobial activity	Nweze and Eze (2009)
2.5–0.0012 mg/ml aminoglycosides + 8–512 μg/ml methanol or hexane extract of *O. gratissimum*	Bacteria	Synergistically inhibited *E. coli* and *S. aureus*	Antimicrobial activity	Matias et al. (2011)
0.0–65.0 mg/mL aqueous extract of *O. gratissimum*	Bacteria	Active against *A. sobria, E. coli,* *P. shigelloides, S. typhi,* and *S. dysenteriae*	Antimicrobial and antidiarrhoeal activities	Ilori et al. (1996)
0.312–40 mg/mL of *O. gratissimum* essential oil	Fungi	Inhibited *F. oxysporum f. sp lycopersici* and *Rhizoctonia solani*	Antifungi	Mohr et al. (2017)
1.0–1000 μg/ml ethanolic crude extract, ethyl acetate, hexane, and chloroformic fractions, essential oil, and eugenol *of O. gratissimum*	Fungi	Chloroformic fraction inhibited 23 isolates (92%) of *C. neoformans* at a concentration of 62.5 μg/ml and eugenol inhibited 4 isolates (16%) at a concentration of 0.9 μg/ml	Antifungi	Lemos et al. (2005)
31.2–1000 μg/ml of hexane, chloroform fractions, the essential oil of *O. gratissimum* extract	Dermatophyte isolates: *M. canis, M. gypseum,* *T. rubrum* and *T. mentagrophytes.*	Hexane and eugenol fractions inhibited the growth of 100% and 80% of dermatophytes respectively, at a concentration of 125 μg/ml)	Antifungal activity	Silva et al. (2005)
0.5, 1, 2, 4, and 8 μg/ml of essential oil of *O. gratissimum* extract	*Candida albicans, Candida krusei, Candida parapsilosis,* *Candida tropicalis*	Fungicidal activity against all of the tested Candida species	Antifungal activity	Nakamura et al. (2004)
100–1000 μg/ml of eugenol-rich essential oil of *O. gratissimum*	Leishmania amazonensis	Inhibited *Leishmania amazonensis*	Anti-leishmanicidal activity	Ueda-Nakamura et al. (2006)
0–200 μg/mL *O. gratissimum* extract	In vitro antioxidant assays	The extract showed potent free radical scavenging activity, and protective effect against lipid, DNA and protein damage	Exhibits antioxidant activity	Venuprasad et al. (2014)
200 and 400 mg/kg of *O. gratissimum* leaf extract	Rats induced intraperitoneally with 50 mg/kg of phenylhydrazine (PHZ) for 2 consecutive days	The extract significantly improved PCV, Hb, and RBC in rats	Anti-anaemic property	Akara et al. (2021)
200 and 400 mg/kg of *O. gratissimum* leaf extract	Rats induced intraperitoneally with 50 mg/kg of phenylhydrazine (PHZ) for 2 consecutive days	The extract reduced the levels of aspartate aminotransferase, alanine aminotransferase, and alkaline phosphatase.	Hepatoprotective effect	Akara et al. (2021)
50 μL concentrations of 20–120 μg/mL of *O. gratissimum* leaf extract	*In vitro*	The extract inhibits ACE and scavenge DPPH in a dose-dependent manner	Management of obesity and obesity-related hypertension	Irondi et al. (2016)
400 mg/kg of aqueous leaf extract of *O. gratissimum*	Diabetic rats (diabetes mellitus was induced using 100 mg/kg of alloxan monohydrate)	Fructosamine and FBG were reduced	Hypoglycaemic effect	Shittu et al. (2019)
208 mg/kg of aqueous leaf extract of *O. gratissimum*	Type 1 diabetic rat model induced with intraperitoneal with a single dose of 65 mg/kg body weight of streptozotocin.	The extract reduced the blood glucose concentration	Hypoglycaemic effect	Okon and Umoren (2017)
400 mg/kg methanolic extract of *O. gratissimum* leaf	Alloxan-induced diabetic rats	Reduced blood sugar level in both normal and diabetic rats by 56 and 69%, respectively.	Hypoglycaemic activity	Aguiyi et al. (2000)
500–1500 mg/kg of aqueous leaf extract of *O. gratissimum*	Streptozotocin induced diabetic rats.	Reduced plasma glucose levels	Hypoglycaemic activity	Egesie et al. (2006)
400 mg/kg extract of *O. gratissimum*	Alloxan monohydrate- induced diabetic rats	Reduced malondialdehyde (MDA) and increased superoxide dismutase (SOD) activities, decreased fasting blood glucose and increased in packed cell volume, red blood cell count, and hemoglobin concentration	Antioxidant activity, improves hematological parameters antidiabetic activity	Shittu et al. (2016)
3 mg/kg chicoric acid from *O. gratissimum*	Streptozotocin-induced diabetic mice	Reduced glycemic levels in diabetic mice	Hypoglycemic activity	Casanova et al. (2014)
250 and 500 mg/kg of methanol and oil extracts of *O. gratissimum*	Male albino rats	No inhibitory effect on the reproductive function and fertility	No detrimental effect reproductive function and fertility	Joseph et al. (2019)
400 mg/kg of aqueous leaf extract of *O. gratissimum*	Diabetic rats (diabetes mellitus was induced using 100 mg/kg of alloxan monohydrate)	Arrested sperm maturation with empty spermatozoa in lumen, and decreased sperm count	Impairs sperm production in diabetic- induced rats	Shittu et al. (2019)
1:10 w/v of *O. gratissimum* leaves	Rats	The extracts had inhibitory effect on phosphodiesterase-5 (PDE-5), angiotensin I –converting enzyme (ACE), acetylcholinesterase (AChE), and arginase	Management of erectile dysfunction	Ojo et al. (2019)
400 and 800 μg/mL leaf extract of *O. gratissimum*	Hepatocellular carcinoma cells	The extract decreased the cell viability of HCC SK-Hep 1 and HA22T cells. It also decreased caspase 3 and PARP expressions, and CDK4and p-ERK1/2 expressions.	Inhibits cell viability and tumor growth	Huang et al. (2020)
50, 100 and 200 mg/kg of *O. gratissimum* leaf extract	Rodents	The extract exhibited antinociceptive and anti-inflammatory effects	Management of painful and inflammatory conditions	Tanko et al. (2008)
0.0125–100 mg/kg of *O. gratissimum*	Rats	The extract inhibited free radicals and suppressed inflammation in carrageenan-induced inflammation	Management inflammation and oxidative stress in chronic diseases	Ajayi et al. (2017a)
25–100 mg/kg of flavonoid-rich fraction of *O. gratissimum* leaves	Lipopolysaccharide-induced mice	The extract attenuates inflammatory and Oxidative Stress in lipopolysaccharide-induced mice	Anti-inflammatory and anti-oxidative stress	Ajayi et al. (2019)
10, 20, or 40 mg/kg of *O. gratissimum* essential oil	Mice	Promoted anti-hypernociception and reduced the levels of interleukin-1β in the sciatic nerve	Anti-hypernociceptive activity	Paula-Freire et al. (2013)
0.005–200 mg/kg of flavonoid-rich fraction of *O. gratissimum*	Peritonitis induced-rats	Reduced neutrophils, monocytes, NO, IL-1β, and TNF-α	Anti-inflammatory activity	Ajayi et al. (2017b)
200, 400 and 800 mg/kg of polyphenol rich extract of *O. gratissimum*	Dextran sodium sulfate (DSS)-induced rat colitis models	Attenuated inflammation, and decreased disease activity index scores in rats with colitis. Decreased Interleukin-(IL)-6 and tumor necrosis factor (TNF)-α, myeloperoxidase, nitric oxide, cyclooxygenase-2 and malondialdehyde in the colon	Repairs colonic mucosa injury via anti-inflammatory and anti-oxidant activity	Alabi et al. (2018)
50 and 100 mg/kg of phenolic-enriched ethylacetate fraction of *O. gratissimum* leaf extract	Rats	Reduced exudate volume, leucocyte count, nitrite, TNF-a, and myeloperoxidase activity. Protected against carrageenan-induced lipid peroxidation and glutathione depletion	Anti-inflammatory and anti-oxidant activity	Ajayi et al. (2017c)
1–100 μg/mL of aqueous and methanol extract of fresh aerial part of *O. gratissimum*	*In vitro*	Inhibited DPPH, hydroxyl and nitric oxide radicals, antioxidant activity	Free radical scavenging activity and antioxidant activity	Mahapatra and Roy (2014)
10.2 mg/mL and 23.2 mg/mL of *O. gratissimum* leaf extract	Rodents	Inhibitory action on pain	Analgesic activity	Aziba et al. (1999)
1–25 μg methanol extract of *O. gratissimum*/ml	*In vitro*	Reduced the levels super oxide anion generation, nicotinamide adenine dinucleotide phosphate (NADPH) oxidase activity, myeloperoxidase (MPO) activity, lipid peroxidation, protein carbonyls, oxidized glutathione levels	Anti-oxidant activity, Decreases free radical generation, lipid and protein damage	Mahapatra et al. (2009)
Chloroform extracts of *O. gratissimum* at 100 mg/kg and 200 mg/kg	Cobalt chloride-induced cardio-renal dysfunction Rats	Reduced the levels of H_2_O_2_ and MDA, expression of caspase 8 and restored GSH levels, GPx, SOD and CAT activities	Antioxidant and pro-apoptotic caspase 8 activities	Akinrinde et al. (2016)
30, 100 and 300 mg/kg of *O. gratissimum* essential oil	Male Swiss mice	Inhibited writhing and inflammation	Antinociceptive activity	Rabelo et al. (2003)
200 and 400 mg/kg of methanol or petroleum ether extract	Adult male Swiss albino mice. Anticonvulsant activity was evaluated using pentylenetetrazol-induced seizure in mice	Increased the latency of tonic and tonic-clonic seizures and death. They also offered 50% protection of treated mice against seizure-induced mortality. The extracts and fraction decreased the frequency of line crossing, center square entries, rearing against a wall and grooming, whereas grooming duration and freezing frequency and duration were increased	Anticonvulsant and anxiolytic-like properties.	Okoli et al. (2010) ,
0.0625, 0.12, 0.25, 0.5 and 1.0% of *O. gratissimum* leaf essential oil and eugenol	*Haemonchus contortus*	Efficiently inhibited eclodibility of *H. contortus* eggs	Anthelmintic activity	Pessoa et al. (2002)
100, 200, and 400 mg/kg/day of *O. gratissimum* leaf	Gentamicin-induced kidney injury in rats	Increased GSH, urine, and plasma creatinine and decreased TBARS, and urine total protein	Management of gentamicin-induced kidney injury	Ogundipe et al. (2017)
150 or 300 mg/kg of ethanol extract of *O. gratissimum*	Focal ischemia and reperfusion (I/R) insult in rat brain.	Attenuated brain oxidative stress, damage and neurological deficits	Neuroprotective effect on cerebral ischemia	Bora et al. (2011)
20–80 mg/mL aqueous *O. gratissimum* extract	Hydrogen peroxide-induced toxicity in human HepG2 cells	Reduced thiobarbituric acid reactive substance (TBARS) formation.	protective effect on oxidative stress in HepG2 cells	Chiu et al. (2012)
0–40 mg/kg *O. gratissimum* extracts	CCl4-induced rats	Reduced liver damage, steatosis and fibrosis. Increased catalase and anti-oxidative enzymes	Anti-hepatic fibrosis properties	Chiu et al. (2014)
12.5–300 μg/mL aqueous extract *O. gratissimum*, hydrophobic and hydrophilic fractions	Human breast comedo-ductal carcinoma in situ	Decreased basement membrane disintegration, angiogenesis and matrix metalloproteinases (MMP-2 and MMP-9) activities	Inhibition of tumor growth and breast cancer cell	Nangia-Makker et al. (2013)
0, 50, 100, 150, and 200 μg/mL of aqueous extract *O. gratissimum*	Schwann RSC96 Cells	Inhibited H_2_O_2_-induced apoptotic protein caspase-3 activation and PARP cleavage, and reversed Bax up-regulation and Bcl-2 down-regulation.	Ameliorates cell stress and stress-induced apoptosis	Chao et al. (2017)
125 and 250 mg/kg/bw ethanol extract *O. gratissimum*	Lead acetate induced Wistar rats	Reduced MDA, increased GSH, SOD, and CAT. Attenuated anemia, thrombocytopenia, and leucocytosis	Anti-oxidant and anti-anaemia activities	Oyem et al. (2021)
200 and 400 mg/kg/day of aqueous leaf extract of *O gratissimum*	Acetic acid-induced colitis in male Wistar rats	Decreased the activities of MPO, SOD, NO and increased GSH levels in colitis rats Decreased diarrhea score and ulcer score to normal	Anti-inflammatory and anti-oxidative properties. Ameliorated colitis	Olamilosoye et al. (2019)
100 or 500 mg/kg BW of *O. gratissimum* (whole plant)	Rats	Reduced systolic blood pressure, ACE levels in plasma and lung, and plasma endothelin-1 at 500 mg/kg dose	Antihypertensive effects	Shaw et al. (2017)
500 mg/kg of 95%of ethanol extract of *O. gratissimum*	Collagen-induced arthritis in rats	Reduced arthritic score and paw volume	Antiarthritic activity	Madhu and Harindran (2014)
10–20 ml/kg aqueous extract of *O. gratissimum*	Castor oil-induced diarrhoea rats	Inhibited castor oil-induced diarrhoea	Antidiarrhoeal effects	Offiah and Chikwendu (1999)
0.2 mg/kg body weight of *O. gratissimum*	carbon tetrachloride (CCl_4_)-induced rats	Decreased stress proteins- (HSP70 and iNOS), MMP-9/MMP-2 ratio, phosphorylated ERK (p-ERK) and NF-κB (p-P65)	Hepatoprotective effect	Chiu et al. (2012)

### 5.2 Anxiolytic activity

Anxiety is a mental disorder marked by a person's unpleasant conduct and inner turmoil that affects people of all ages, from children to the elderly. Benzodiazepines and other allopathic medicines that non-selectively target gamma-aminobutyric acid (GABA) receptors are commonly used to treat anxiety (Rudolph and Knoflach, 2011). Okoli et al. (2010) reported that 200 and 400 mg/kg of methanol or petroleum ether extract increased the latency of tonic and tonic-clonic seizures and death. They also offered 50% protection in treated mice against seizure-induced mortality. Several studies have shown a link between antioxidant activity and anti-anxiety action (Hovatta et al., 2010). Several gene products play an important role in anxiety, with glyoxalase-1 and glutathione-1 being the major proteins whose activities affect anxiety (Hovatta et al., 2005). Venuprasad et al. (2014) emphasized *O*. *gratissimum's* protective effect against DNA, protein, and lipid peroxidation. Pre-treatment with the leaf extract of the plant resulted in 44.8% protection from H_2_O_2_-induced DNA damage in SH-SY5Y human neuronal cells, 80% protection from AAPH-induced BSA oxidation, and lipid peroxidation inhibition at an IC_50_ value of 735 g/mL. As observed from the open field and elevated plus maze tests, the plant had a substantial anxiolytic effect on the test mice at a dosage of 400 mg/kg body weight (Venuprasad et al., 2014).

### 5.3 Antinociceptive activity

In traditional medicine, *O*. *gratissimum* is used to treat painful conditions. In classic pain models, the antinociceptive effects of *O*. *gratissimum* essential oil and two of its active components (eugenol and myrcene) were investigated (hot plate test and formalin test) on neurogenic and inflammatory pain in murine pain models (Paula-Freire et al., 2013). In the first and second stages of the formalin test, the essential oil of *O*. *gratissimum* at a dose of 40 mg/kg, as well as its active components, eugenol and myrcene, at a dose of 10 mg/kg, successfully reduced pain in animals. These findings support the work of other authors on the antinociceptive action of the essential oil of *O*. *gratissimum* (Paula-Freire et al., 2013). Rabelo et al. (2003) showed that 30, 100 and 300 mg/kg of *O. gratissimum* essential oil inhibited writhing and inflammation in male Swiss mice. The report of Tanko et al. (2008) showed a significant antinociceptive effect of *O*. *gratissimum* at 1264.9 mg/kg body weight in the acetic-acid-induced abdominal constriction test and hot plate technique in rats, and this supports its traditional use in pain management.

### 5.4 Neuroprotective activity

Supplements of *O*. *gratissimum* leaf extract promote brain performance. Ajayi et al. (2018) reported the use of *O. gratissimum* at body weights of 25, 50, and 100 mg/kg in the treatment of behavioural impairment and depressive-like behaviour in mice using the open field test (OFT) and the forced swim test (FST). Bora et al. (2011) revealed that the neuroprotective ability of the extract of *O*. *gratissimum* in cerebral ischaemia is mediated by its antioxidant properties as observed during pre-treatment in Wistar rats with middle cerebral artery occlusion for 24 h followed by 24-hour reperfusion, as shown in Table 3.

### 5.5 Antimicrobial activity

A couple of studies have confirmed the antimicrobial activities of *Ocimum gratissimum* (Ilori et al., 1996; Nweze and Eze, 2009; Prakash et al., 2011; Melo et al., 2019). Prakash et al. (2011) described *Ocimum gratissimum* essential oil as a plant-based preservative and suggests its use as a nontoxic antibacterial and anti-aflatoxigenic agent against fungal and aflatoxin contamination of spices and as a shelf-life enhancer due to its antioxidant activity. Chimnoi et al. (2018) showed that 0.015–8.00 mg/ml essential oil extract of *O. gratissimum* leaf caused rapid inhibition of *Escherichia coli* and *S. typhimurium*. As reported by Talabi and Makanjuola et al. (2017), the aqueous extract of *O*. *gratissimum* strongly inhibited *Pseudomonas aeruginosa* and moderately inhibited *Staphylococcus aureus*, but the aqueous ethanolic leaf powder extract demonstrated a broader range of antimicrobial activities with significant inhibitory properties against *E. coli*, *Bacillus cereus, P*. *aeruginosa*, and *S*. *aureus* (Talabi and Makanjuola, 2017). Joshi (2013) used the tube-dilution method to test the antibacterial activity of *O*. *gratissimum* essential oils and its primary ingredient, eugenol, revealed strong antibacterial activity against *Klebsiella pneumoniae*, *Serratia marcescens*. and *E*. *coli*. Matias et al. (2011) reported that 2.5–0.0012 mg/ml aminoglycosides and 8–512 μg/ml methanol or hexane extracts of *O*. *gratissimum* synergistically inhibited *E. coli* and *S. aureus*.

Many studies have reported the antifungal activities of *O*. *gratissimum* (Lemos et al., 2005; Mohr et al., 2017). Lemos et al. (2005) showed that chloroformic fraction inhibited 23 isolates (92 %) of *C. neoformans* at a concentration of 62.5 μg/ml and eugenol inhibited 4 isolates (16 %) at a concentration of 0.9 μg/ml Mohr et al. (2017) revealed that 0.312–40 mg/mL of *O. gratissimum* essential oil inhibited *F*. *oxysporum* f. sp lycopersici and *Rhizoctonia solani*. Nakamura et al. (2004) investigated the *in vivo* antifungal activity of *O*. *gratissimum* essential oil against several *Candida* species. They were able to demonstrate that the plant extract had fungicidal activity against four distinct species of *Candida* examined, namely *Candida albicans*, *Candida Krusei*, *Candida parapsilosis*, and *Candida tropicalis*, by measuring their minimum inhibitory concentrations and time curves. The bud development of fungal cells treated with the extract was impaired. Significant alterations occurred in the cell wall and the structure of the subcellular organelles. These findings indicate that *O*. *gratissimum* essential oil might possibly be used as a phytotherapeutic agent in the treatment of various fungal disorders, and as a fungicidal agent in the management of fungi in the environment (Nakamura et al., 2004). According to Silva et al. (2005), the leaf extract of the plant contains bioactive components that are effective *in vitro* against dermatophytes. These findings further support *O*. *gratissimum's* antifungal activity.

Nakamura et al. (1999) conducted an experimental examination of the antibacterial activity of *O*. *gratissimum* essential oil on various bacterial species. The essential oil of *O*. *gratissimum* extract suppressed the growth of *S*. *aureus* at 0.75 μg/mL concentration. Minimal inhibitory concentrations (MICs) ranged from 3 to 12 μg/mL for *Shigella flexineri*, *Salmonella enteritidis*, *E*. *coli*, *Klebsiella* species, and *Proteus mirabilis*. Eugenol was discovered as the primary component responsible for the antibacterial action of *O*. *gratissimum* essential oil. As a result, the plant has the potential to be used therapeutically in the prevention and treatment of bacterial infections (Nakamura et al., 1999).

Melo et al. (2019) investigated the antibacterial activity of *O*. *gratissimum* essential oil extract against multidrug-resistant bacteria in planktonic and biofilm forms, such as isolates of *S*. *aureus* and *E*. *coli*. The antibacterial activity of the essential oil extract was determined by disk diffusion, while the checkerboard assay was used to assess the microdilution (MIC/MBC), growth curve under sub-MIC exposure, and combinatorial activity with ciprofloxacin and oxacillin. *S*. *aureus* and *E*. *coli* had much lower biofilm biomass and cell viability. These findings support the use of *O*. *gratissimum* essential oil extract as a natural option for treating infections caused by multidrug-resistant bacterial strains (Melo et al., 2019). Aneke et al. (2019) reported that except for *Penicillium* species, two primary dermatomycotic agents isolated from animals exhibited considerable activity at 128 μg/mL concentration of ethanolic leaf extract of *O*. *gratissimum*, namely *Microsporum* species and *Trichophyton* species. They showed a considerable degree of susceptibility at 128 μg/mL concentration. These findings imply that ethanolic extracts of *O*. *gratissimum* contain active components with antifungal action against these fungal pathogens. As a result, it has the potential to be employed therapeutically in the treatment of dermatomycoses (Aneke et al., 2019) (Table 3).

### 5.6 Anti-protozoal activity

The essential oils and ethanolic crude extract of *O*. *gratissimum* leaves and stems were evaluated *in vitro* against *Trypanosoma brucei* and *Plasmodium falciparum* in pre- and full flowering phases. The best growth inhibition of *Trypanosoma brucei* was observed, suggesting its antiprotozoal activities (Kpoviessi et al., 2014). In an *in vitro* and *in vivo* study reported by Adamu et al. (2009), the survival time of the parasite (*Trypanosoma brucei*) was dependent on the concentration of the extract, with lower (25 and 12.5 mg/ml) concentrations lasting longer than higher (100, 75, and 50 mg/mL) concentrations.

Tchoumbougnang et al. (2005) conducted a study on the suppressive impact of *O*. *gratissimum* essential oil extract on *Plasmodium berghei* development *in vivo* in an animal model. According to the findings of their investigation, the extracted essential oil of *O*. *gratissimum* shows considerable antimalarial activity in test mice after a four-day suppressive *in vivo* assessment. The plant's essential oil extract showed highest effectiveness at dosages of 200, 300, and 500 mg/kg, with parasitaemia suppression percentages of 62.1 %, 81.7 %, and 86.6 %, respectively. Based on the findings of this investigation, the essential oil extract of *O*. *gratissimum* might be therapeutically effective in the treatment and control of malaria (Tchoumbougnang et al., 2005) (Table 3).

### 5.7 Anti-anaemic activity

Several studies have suggested that *O*. *gratissismum* leaf extract has the ability to abate toxicities induced on haematological indices of Wistar rats. For example, Akara et al. (2021) reported that *O*. *gratissimum* leaf extract at the dose of 400 mg/kg body weight ameliorates phenylhydrazine-induced anaemia in rats. Extracts of *O*. *gratissimum* were identified to have haematological effects on the body as they led to an increase in the red blood cells, packed cell volume, haemoglobin, and platelet and neutrophil counts. A decrease in the platelet count was observed when 500 mg/kg of the extract alongside feed pellets were used (Ofem et al., 2012). Akara et al. (2021) contended that the iron and vitamins present in the aqueous extract of *O*. *gratissimum* may be responsible for the haematopoietic characteristics found in their investigation (Table 3).

### 5.8 Bio-pesticide

Essential oils from *Ocimum gratissimum* at a concentration of 1 μL/mL showed repellence and a toxic fumigation effect when used on *Tuta absoluta* (Essoung et al., 2020). The adult form of *Tribolium castaneum* is susceptible to the cinnamic acid esters isolated from *O*. *gratissimum* at 26.92 mg/mL concentration (Buxton et al., 2020). Nguemtchouin et al. (2013) revealed that a mortality rate of 100–95% was observed with the combination of modified montmerillonite clay and *O*. *gratissimum* essential oil, which declined after 30 days as it had lost its insecticidal function by 60% and persisted for around 7–80 days.

### 5.9 Wound healing properties

Chang et al. (2021) reported that 100 μg/mL *O. gratissimum* restored cell activity and protected against ultraviolet C-induced inhibition of cell proliferation and migration of skin cells, and therefore can serve as a potent natural wound care agent. Orafidiya et al. (2006) state that the formulation containing 2% *O*. *gratissimum* and honey as a surfactant had an antibacterial effect on *S*. *aureus*. It indicated that the net electrical charge on the surfactant with which it is produced influences the antibacterial activity of ocimum oil. The remarkable antibacterial action of the 2% ocimum oil in honey formulation, together with the documented wound healing characteristic of honey, suggests that the 2% ocimum oil in honey formulation might be useful as a topical antiseptic agent for wounds (Orafidiya et al., 2006).

### 5.10 Enzyme-inhibitory activity

The leaf extract of *O*. *gratissimum* inhibited pancreatic lipase and angiotensin 1-converting enzyme significantly, with IC_50_ values of 20.69 g/mL and 29.44 g/mL, respectively. As a result, the leafy parts of *O*. *gratissimum* can be used as functional foods in a therapeutic approach for the control of obesity and obesity-related hypertension (Irondi et al., 2016). Ojo et al. (2019) investigated the inhibitory effect of an aqueous extract (1:10 w/v) of *O*. *gratissimum* leaves on some vital enzymes associated with erectile dysfunction, such as phosphodiesterase-5 (PDE-5), arginase, angiotensin 1-converting enzyme (ACE), and acetylcholinesterase (AChe), in penile and testicular tissues of study rats. The results of the study revealed that the extract has inhibitory effects on enzyme activities linked with erectile function, as well as free radical scavenging capacities, and these activities are linked with the plant's phenolic and flavonoid components (Ojo et al., 2019) (Table 3).

### 5.11 Analgesic activity

Aziba et al. (1999) studied the pharmacological activity of *O*. *gratissimum* aqueous extracts in isolated rabbit jejunum, as well as their analgesic qualities in mice. Following administration of the extract, the spontaneous pendular movement of the rabbit jejunum was inhibited in a dose-dependent manner. The analgesic investigation also indicated an extended reaction time of 85% throughout a 20-minute observation period with no evident signs of toxicity. Their findings suggested that the aqueous extract of *O*. *gratissimum* has analgesic and spasmolytic properties (Aziba et al., 1999). Ajayi et al. (2017b) reported that *O. gratissimum* exhibited the potential to reduce pain perception in analgesic experiments, as observed when all the constituents of *O. gratissimum* were administered to mice.

### 5.12 Larvicidal activity

*O*. *gratissimum* substantially reduced the mortality of *Anopheles gambiae* larvae at all doses of the extract tested on the organisms in an experiment conducted by Ileke and Adesina (2019). It had a remarkable effectiveness against the pre-adult stages and adults of *Anopheles gambiae*, leading to 90% pupae death at a concentration of 0.5% (Ileke and Adesina, 2019). Following exposure of newly emerging adult beetles (*Callosobruchus maculatus*) at a dose of 25 mL/vial during a fumigation operation, essential oils produced by steam distillation from *O*. *gratissimum* caused 80% death. It also had a substantial influence on the egg hatch rate, lowering it to roughly 15% at a concentration of 30 mL for the extract. Thus, the essential oil of *O*. *gratissimum* may have anti-larval characteristics that might be useful in pest management (Keita et al., 2001). Harikarnpakdee and Chuchote (2018) reported that *O*. *gratissimum* has the potential to enhance the control of dengue fever. They investigated the oviposition deterrent activity of the essential oil of *O*. *gratissimum* and *O*. *gratissimum* alginate beads against *Aedes aegypti* (*Ae. aegypti*) mosquitoes. The study's findings revealed that the beads offered substantially longer oviposition deterrent efficacy against gravid *Aedes aegypti*, but free *O*. *gratissimum* oil only did so for a shorter amount of time. The results suggest a less expensive method of managing dengue disease (Harikarnpakdee and Chuchote, 2018).

### 5.13 Leishmanicidal activity

Ueda-Nakamura et al. (2006) found that the eugenol-rich essential oil of *O*. *gratissimum* had increasing inhibitory actions against *Leishmania amazonensis* growth at extract concentrations ranging from 100 to 1000 Ag/mL. At inhibitory concentrations (IC_50_) of 135 and 100 Ag/ml for promastigotes and amastigotes, respectively, there were significant mitochondrial alterations in essential oil-treated promastigotes and amastigotes. Both promastigotes and amastigotes have a minimum inhibitory concentration of 150 Ag/ml. Their research revealed that the essential oil had no cytotoxic effects on mammalian cells. This underlines the potential effectiveness of the essential oil of *O*. *gratissimum* against protozoas and as a novel therapeutic source of antileishmanial drugs (Ueda-Nakamura et al., 2006).

### 5.14 Ovicidal activity

Pessoa et al. (2002) conducted a research to assess the ovicidal efficacy of *O*. *gratissimum* essential oil and its major component, eugenol, against *Haemonchus contortus*, a gastrointestinal parasite found in small ruminants. During the egg hatch test, *H*. *contortus* eggs were also retrieved from the faeces of goats that had been experimentally infected with the test organism. The essential oil and eugenol, on the other hand, revealed a maximum eclodibility suppression of the organism's growth at 0.5% concentration. These findings suggest that the use of the essential oil of *O*. *gratissimum* might be a useful method to manage gastrointestinal helmintosis in small ruminants (Pessoa et al., 2002).

### 5.15 Cytotoxic activity

The cytotoxic impact of methanolic extract *O*. *gratissimum* (ME-Og) was studied in murine peritoneal macrophages at doses ranging from 0.1 to 100 g/ml using the 3-(4,5-dimethylthiazol-2-yl)-2,5 diphenyltetrazolium bromide (MTT) technique. The findings indicated that *O*. *gratissimum* plant extracts have a considerable modulatory influence on nicotine-induced free radical production, lipid-protein damage, and antioxidant status in peritoneal macrophages. Following a 12-hour treatment of murine peritoneal macrophages in culture medium with hazardous 10 mM nicotine, it was observed that ME-Og exerted a protective effect against nicotine toxicity. In rats treated with the extract, the impact of the poisonous substance was significantly decreased and the negative effects were significantly reduced. Therefore, the results confirm the modulatory effect of *O*. *gratissimum* on hazardous chemicals such as nicotine (Mahapatra et al., 2009).

Chiu et al. (2012) found that *O*. *gratissimum* aqueous extract (OGAE) protected the liver against CCl_4_-induced damage in rats utilized as a model of chronic hepatic damage. An increase in the blood catalase activity of CCl_4_-administered rats treated with the aqueous plant extract was observed, which was much higher than that of the control rats treated with saline solution. In a study comparing those given OGAE to those given saline or CCl_4_, it was observed that the livers of the CCl_4_-administered rats given the plant extract showed a significant decrease in stress proteins such as heat shock protein (*HSP70*) and inducible nitric oxide synthase (iNOS). A significant reduction in the ratio of MMP-9/MMP-2, phosphorylated ERK (*p*-ERK), and NF-kB (*p*-P65) was observed, suggesting the possible protective effect of OGAE against CCl_4_-induced toxicity in rats (Chiu et al., 2012).

The aqueous extract of *O*. *gratissimum* exhibited ameliorative activities, which enabled it to provide protective benefits against acetic acid-induced colitis in male rats when administered at doses ranging from 200 to 400 mg/kg/day for 20 consecutive days (Olamilosoye et al., 2019). Similarly, it ameliorated the haematological parameters and significantly reduced the activities of MPO, SOD, and NO and increased the GSH levels in colitis rats (Olamilosoye et al., 2019). *O*. *gratissimum* ameliorated the cytotoxic effects of H_2_O_2_-induced apoptosis by modulating the apoptotic pathway at concentrations ranging from 150 to 200 μg/mL (Chao et al., 2017) (Table 3).

### 5.16 Anti-infertility effect

When administered, *Ocimum gratissimum* was observed to have an effect on penile and testicular tissues present in rats in the treatment of erectile dysfunction (Ojo et al., 2019). Joseph et al. (2019) reported that methanolic and oil extracts of *O*. *gratissimum* leaves, when administered at two dosages of 250 and 500 mg for 14 and 28 days, had no negative effect on the reproductive capabilities of the male rats.

### 5.17 Hepatoprotective activity

Ajayi et al. (2011) discovered that *O*. *gratissimum* given to mice at different concentrations might protect the liver by controlling the increase in catabolic enzyme levels induced by CCl_4_ damage in liver cells. Chiu et al. (2014) opined that 0–40 mg/kg *O. gratissimum* extracts reduced liver damage, steatosis, and fibrosis and increased catalase and anti-oxidative enzymes. Huang et al. (2020) revealed that in hepatocarcinomal cell (HCC) following treatment with *O*. *gratissimum* at concentrations more than 20 mg/mL, an increase in p/ERK1/2 levels was observed, indicating that it has an effect on the survival signalling of the liver cancer cells. The flavonoid-rich ethyl acetate fraction of *O*. *gratissimum* was discovered to exhibit hepatoprotective properties, indicating that it may be useful in slowing down the LPS-mediated sickness process in mice with LPS-induced sickness behaviour (Ajayi et al., 2019). Fandohan et al. (2008) reported that the administration of *O. gratissimum* oil at the dose of 1000 mg/kg did not produce any hepatotoxic effects on the rat's liver, as shown in Table 3.

### 5.18 Nephroprotective activity

Ogundipe et al. (2017) showed that 100, 200, and 400 mg/kg/day of *O*. *gratissimum* leaf could be employed in the management of gentamicin-induced kidney injury. Treatment with *O*. *gratissimum* aqueous leaf extract, on the other hand, induced a reduction in creatinine, urea, HCO_3_, K^+^, Cl^−^, and Na^+^, suggesting that *O*. *gratissimum* leaf extract may have renoprotective properties (Akara et al., 2021).

### 5.19 Anti-diarrhoeal activity

Offiah and Chikwendu (1999) investigated the anti-diarrhoeal effects of an aqueous extract of *O*. *gratissimum* leaves. They were able to demonstrate the anti-diarrhoeal efficacy of the extract on castor oil-induced diarrhoea in rats, as evidenced by a substantial decrease in the amount of wet faeces in rats treated with the extract. The aqueous leaf extract of the plant reduced diarrhoea and the propulsive movement of intestinal contents. It also had no direct effect on guinea pig harvested ileum, but it greatly reduced the ileum's responsiveness to acetylcholine, nicotine, and histamine, resulting in lower contractile activity induced by these drugs. Further phytochemical assessments revealed that the plant's principal constituents include tannins, steroids, triterpenoid, and carbohydrates. These findings suggest that the aqueous extract of *O*. *gratissimum* leaves may include pharmacologically active ingredients with antidiarrhoeal effects defined primarily by inhibitory activity on intestinal motility, perhaps *via* muscarinic receptor inhibition (Offiah and Chikwendu, 1999), as shown in Table 3.

### 5.20 Anti-diabetic activity

Studies on the hypoglycaemic activities of *O*. *gratissimum* have been reported by various researchers using animal models (Egesie et al., 2006; Shittu et al., 2019). In mice, co-administration of the *O*. *gratissimum* leaf extract after oral administration of starch and glucose demonstrated that the extract inhibited the increase in postprandial blood glucose levels (Shimada et al., 2019). Their findings suggested that the inhibitory effect on sodium-dependent glucose transporter (*SGLT1*) could be one of the underlying mechanisms of the anti-hyperglycaemic effect of the leaf extract of *O*. *gratissimum* (Shimada et al., 2019). Aguiyi et al. (2000) observed a significant reduction in plasma glucose levels when *O*. *gratissimum* was administered at 400 mg/kg body weight. The research carried out by Casanova et al. (2014) showed the hypoglycaemic activity of *O*. *gratissimum* against streptozotocin-induced diabetes in rats. They contended that the chicoric acid, a major phenolic constituent, may be responsible for this activity. However, further characterization of the plant's bioactive components is recommended to determine other bioactive components that may exhibit these pharmacological activities. Other studies reported by Antora and Salleh (2017) showed that extracts of *O. gratissimum* when used on streptozotocin-induced diabetic rats at 500 mg/kg was found to be 81% effective. Okon and Umoren (2017) contended that the hypoglycaemic efficacy of *O*. *gratissimum* was higher than that of insulin in streptozotocin-induced diabetic rats at 208 mg/kg (Table 3).

### 5.21 Anti-inflammatory activity

The plant has been reported to exhibit anti-inflammatory activities (Ajayi et al., 2017a,b,c; Alabi et al., 2018). For example, Ajayi et al. (2017a) suggested that *O*. *gratissimum* extract when used to treat rats induced with carrageenan was able to reduce inflammations at 50,100, and 200 mg/kg body weight. The extract at 100 mg/kg body weight had a significant effect on rats by inhibiting carrageenan-induced paw oedema, suggesting its therapeutic use in the treatment of inflammations (Ajayi et al., 2019). In the study conducted by Alabi et al. (2018), *O*. *gratissimum* was found to have anti-inflammatory effects on dextran sodium sulphate (DSS) induced colitis in rats at doses of 100–800 mg/kg where signs of repair were evident. The extract was found to be useful in the treatment of eosinophilic airway inflammation in male AJ mice induced by *Blomia tropicalsis*. In a murine model, doses of 25, 50, and 100 mg/kg of methanolic extract of the plant were found to be effective in alleviating respiratory allergy (Costa et al., 2012) (Table 3).

### 5.22 Anti-hypertensive activity

*O*. *gratissimum* at 100 and 200 mg/kg improved blood pressure and toxic processes in cobalt chloride-induced cardiorenal dysfunction in rats (Akinrinde et al., 2016). Shaw et al. (2017) investigated the inhibitory effect of *O*. *gratissimum* (8 weeks at 100 or 500 mg/kg) on angiotensin-converting enzyme (ACE) in hypertensive rats (Table 3).

### 5.23 Anticancer activity

Anticancer activities from plant bioactive components have been documented (Sun et al., 2002; Surh, 2003; Ohiagu et al., 2021). Lin et al. (2014) investigated the anticancer efficacy of an aqueous extract of *O*. *gratissimum* due to its antioxidant capabilities after treating human osteosarcoma cells with the extract, adding to the expanding body of research on the plant and its involvement in cancer treatments. Cell viability experiments demonstrated that the activity of the aqueous extract of *O*. *gratissimum* affected the viability of U2-OS and HOS cells considerably and dose dependently. It is characterized by an increase in cell shrinkage, sub-G1 fragmentation, and caspase 3 activations (Lin et al., 2014). Treatment with *O*. *gratissimum* at 200 mg/kg caused tumour growth decrease through modulation of the ERK signalling pathway and aerobic glycolysis, and increasing cell apoptosis in mice induced with mahlavu cells (Huang et al., 2020). Nangia-Makker et al. (2013) showed that 12.5–300 μg/mL extract of *O. gratissimum* decreased basement membrane disintegration, angiogenesis, and matrix metalloproteinases (MMP-2 and MMP-9) activities. This subsequently resulted in the inhibition of tumour growth and breast cancer cells. When human breast cancer cells were treated with both *O. basillium* and *O. gratissimum*, it was observed that *O*. *gratissimum* had a lower cystotic and apoptotic impact on the MCF-7 human breast cancer cell line through activation of the mTOR/Akt/AMPK signalling pathway (Torres et al., 2018). Ekunwe and his co-researchers reported that partially purified *O. gratissimum* fractions (Ekunwe et al., 2010) or aqueous or organic solvent-soluble extracts of *O. gratissimum* (Ekunwe et al., 2013) inhibited the proliferation of several cancer cell lines, especially prostate adenocarcinoma (PC-3) cells (Table 3).

### 5.24 Immunomodulatory activity

Mahapatra et al. (2011) investigated the immunological functions and immunological responses in nicotine-induced (10 mM) macrophages as well as the immunomodulatory activity of *O*. *gratissimum* extract. Nicotine-induced NO production and iNOS II expression were considerably reduced after the administration of a 10 μg/mL aqueous extract of the plant. The aqueous extract of the plant had protective effects on murine peritoneal macrophages by downregulating Th1 cytokines in nicotine-treated macrophages while simultaneously activating Th2 responses (Mahapatra et al., 2011).

## 6 Conclusion and future perspectives

In conclusion, *O. gratissimum* has remarkable dietary and pharmaceutical applications, which make it an excellent functional ingredient for use in the treatment of a plethora of health abnormalities. Currently, various researchers are conducting in-depth studies on the useful components of this health-promoting plant. Because *O*. *gratissimum* is an excellent source of vital phytochemicals, nutrients, and essential oils, bioactive isolates with a higher biological value of this plant could be a better replacement for traditional medicine in the treatment of microbial infections, cough, cancer, diarrhoea, anaemia, and inflammatory diseases. Apart from its bioactive potential, *O. gratissimum* is an excellent source of micronutrients. The reported pharmacological/clinical activities exhibited by *O. gratissimum* are not limited to its antioxidant properties and ability to suppress inflammatory biomarkers. The presence of bioactive substances demonstrates the variations in this plant's components. These differences arise as a result of variations in topography, meteorological conditions, yield, preparation process, and many other factors. Therefore, there is a need to investigate the phytochemical properties of *O*. *gratissimum*, which may be utilized to enhance the health benefits (animal and human nutrition), as well as the environment, by using its isolated chemicals in natural weed and pest control management. Therefore, further research in human clinical trials is recommended to effectively confirm the safe concentration of the extract required to manage health deviations. Apart from these benefits, *O*. *gratissimum* has been effective against diabetes, anemia, infertility, diarrhoea, and inflammatory disorders. Hence, further studies are required to determine the specific mechanism by which *O*. *gratissimum* protects against various diseases, which can be used to design and create effective therapies for these disorders in the future. The studies included in this review collectively suggest that this plant has several healing properties. Owing to its multidirectional activities, *O*. *gratissimum* has been regarded as an important medicine for a variety of diseases.

## Declarations

### Author contribution statement

All authors listed have significantly contributed to the development and the writing of this article.

### Funding statement

This research did not receive any specific grant from funding agencies in the public, commercial, or not-for-profit sectors.

### Data availability statement

Data included in article/supplementary material/referenced in article.

### Declaration of interests statement

The authors declare no conflict of interest.

### Additional information

No additional information is available for this paper.
